# Bis{2-[3-(dimethyl­amino)propyl­imino­meth­yl]-4,6-disulfanylphenolato}cobalt(II)

**DOI:** 10.1107/S1600536809031389

**Published:** 2009-08-15

**Authors:** Hai-Yan Li, Li-Jun Wang, Qiang Wang, Qing-Fu Zeng

**Affiliations:** a Engineering Research Center for Clean Production of Textile Dyeing and Printing, Ministry of Education, Wuhan 430073, People’s Republic of China

## Abstract

In the title mononuclear complex, [Co(C_12_H_17_N_2_OS_2_)_2_], the Co^II^ atom is four-coordinated by two *N*,*O*-bidentate Schiff base ligands, resulting in a slightly distorted *trans*-CoN_2_O_2_ square-planar coordination.

## Related literature

For background to Schiff bases, see: Shi *et al.* (2008 ▶); Xu *et al.* (2009 ▶). For reference structural data, see: Allen *et al.* (1987 ▶).
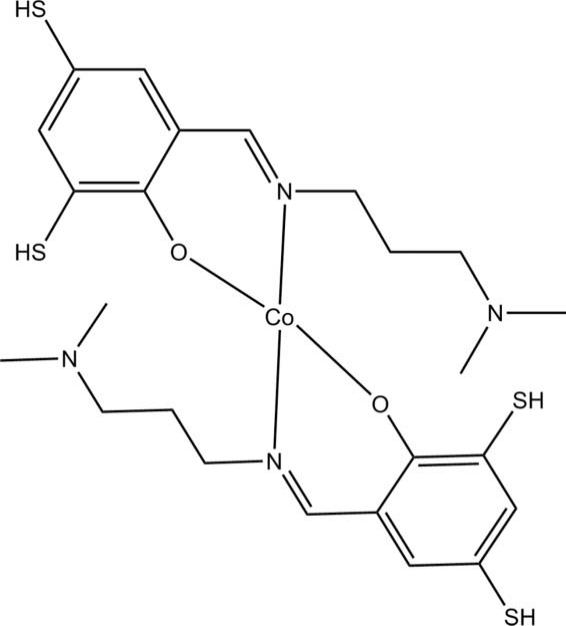


## Experimental

### 

#### Crystal data

[Co(C_12_H_17_N_2_OS_2_)_2_]
*M* *_r_* = 597.72
Triclinic, 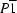

*a* = 9.4100 (18) Å
*b* = 12.5482 (18) Å
*c* = 12.6032 (18) Å
α = 103.272 (8)°
β = 110.910 (8)°
γ = 90.612 (8)°
*V* = 1346.0 (4) Å^3^
*Z* = 2
Mo *K*α radiation
μ = 0.98 mm^−1^
*T* = 296 K
0.40 × 0.30 × 0.30 mm

#### Data collection

Enraf–Nonius CAD-4 diffractometer
Absorption correction: ψ scan (North *et al.*, 1968 ▶) *T*_min_ = 0.696, *T*_max_ = 0.758
7433 measured reflections
5221 independent reflections
3718 reflections with *I* > 2σ(*I*)
*R*_int_ = 0.022

#### Refinement

*R*[*F* ^2^ > 2σ(*F* ^2^)] = 0.042
*wR*(*F* ^2^) = 0.129
*S* = 1.02
5221 reflections
324 parameters
H-atom parameters constrained
Δρ_max_ = 0.43 e Å^−3^
Δρ_min_ = −0.58 e Å^−3^

### 

Data collection: *CAD-4 Software* (Enraf–Nonius, 1989 ▶); cell refinement: *CAD-4 Software*; data reduction: *XCAD4* (Harms & Wocadlo, 1995 ▶); program(s) used to solve structure: *SHELXS97* (Sheldrick, 2008 ▶); program(s) used to refine structure: *SHELXL97* (Sheldrick, 2008 ▶); molecular graphics: *SHELXTL* (Sheldrick, 2008 ▶); software used to prepare material for publication: *SHELXTL*.

## Supplementary Material

Crystal structure: contains datablocks global, I. DOI: 10.1107/S1600536809031389/hb5029sup1.cif

Structure factors: contains datablocks I. DOI: 10.1107/S1600536809031389/hb5029Isup2.hkl

Additional supplementary materials:  crystallographic information; 3D view; checkCIF report

## Figures and Tables

**Table 1:** Selected bond lengths (Å)

Co1—O2	1.922 (2)
Co1—O1	1.925 (2)
Co1—N1	2.003 (3)
Co1—N3	2.011 (2)

**Fig. 1.**
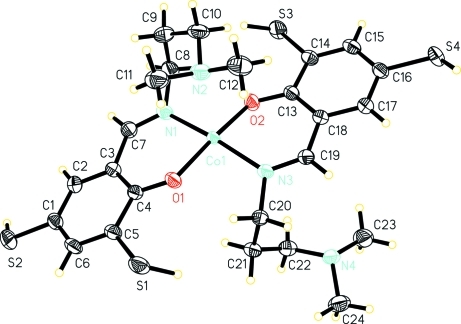
The molecular structure of showing 30% probability displacement ellipsoids.

## supplementary crystallographic information

### Comment

There has been much research interest in Schiff base metal complexes due to their molecular architectures and biological activities (Shi *et al.*, 2008; Xu *et al.*, 2009). In this work, we report here the crystal structure of the title compound, (I). In (I), all bond lengths are within normal ranges (Allen *et al.*, 1987) (Fig. 1). The Co(II) is four-coordinated in a slight distort square-planar configuration by two N atoms and two O atoms of the Schiff base ligand.

### Experimental

A mixture of 2-hydroxy-3,5-disulfanylbenzaldehyde (372 mg, 2 mmol), *N*,*N*-dimethylpropane-1,3-diamine (204 mg, 2 mmol) and CoCl_2_.6H_2_O (1 mmol, 238 mg) was stirred in methanol (10 ml) for 1 h. After keeping the filtrate in air for 7 d, red block-shaped crystals of (I) were formed.

### Refinement

All H atoms were positioned geometrically (C—H = 0.93–0.96Å, S—H = 1.20Å) and refined as riding, with *U*_iso_(H) = 1.2*U*_eq_(carrier) or 1.5U_eq_(methyl C).

### Crystal data

**Table:**

[Co(C_12_H_17_N_2_OS_2_)_2_]	*Z* = 2
*M**_r_* = 597.72	*F*(000) = 626
Triclinic, *P*1	*D*_x_ = 1.475 Mg m^−^^3^
Hall symbol: -P 1	Mo *K*α radiation, λ = 0.71073 Å
*a* = 9.4100 (18) Å	Cell parameters from 25 reflections
*b* = 12.5482 (18) Å	θ = 9–12°
*c* = 12.6032 (18) Å	µ = 0.98 mm^−^^1^
α = 103.272 (8)°	*T* = 296 K
β = 110.910 (8)°	Block, red
γ = 90.612 (8)°	0.40 × 0.30 × 0.30 mm
*V* = 1346.0 (4) Å^3^	

### Data collection

**Table:**

Enraf–Nonius CAD-4 diffractometer	5221 independent reflections
Radiation source: fine-focus sealed tube	3718 reflections with *I* > 2σ(*I*)
graphite	*R*_int_ = 0.022
ω/2θ scans	θ_max_ = 26.0°, θ_min_ = 1.7°
Absorption correction: ψ scan (North *et al.*, 1968)	*h* = −11→9
*T*_min_ = 0.696, *T*_max_ = 0.758	*k* = −15→15
7433 measured reflections	*l* = −15→15

### Refinement

**Table:**

Refinement on *F*^2^	Primary atom site location: structure-invariant direct methods
Least-squares matrix: full	Secondary atom site location: difference Fourier map
*R*[*F*^2^ > 2σ(*F*^2^)] = 0.042	Hydrogen site location: inferred from neighbouring sites
*wR*(*F*^2^) = 0.129	H-atom parameters constrained
*S* = 1.02	*w* = 1/[σ^2^(*F*_o_^2^) + (0.0746*P*)^2^ + 0.0983*P*] where *P* = (*F*_o_^2^ + 2*F*_c_^2^)/3
5221 reflections	(Δ/σ)_max_ = 0.044
324 parameters	Δρ_max_ = 0.43 e Å^−^^3^
0 restraints	Δρ_min_ = −0.58 e Å^−^^3^

### Special details

**Table:**

Geometry. All e.s.d.'s (except the e.s.d. in the dihedral angle between two l.s. planes) are estimated using the full covariance matrix. The cell e.s.d.'s are taken into account individually in the estimation of e.s.d.'s in distances, angles and torsion angles; correlations between e.s.d.'s in cell parameters are only used when they are defined by crystal symmetry. An approximate (isotropic) treatment of cell e.s.d.'s is used for estimating e.s.d.'s involving l.s. planes.
Refinement. Refinement of *F*^2^ against ALL reflections. The weighted *R*-factor *wR* and goodness of fit *S* are based on *F*^2^, conventional *R*-factors *R* are based on *F*, with *F* set to zero for negative *F*^2^. The threshold expression of *F*^2^ > σ(*F*^2^) is used only for calculating *R*-factors(gt) *etc*. and is not relevant to the choice of reflections for refinement. *R*-factors based on *F*^2^ are statistically about twice as large as those based on *F*, and *R*- factors based on ALL data will be even larger.

### Fractional atomic coordinates and isotropic or equivalent isotropic displacement parameters (Å^2^)

**Table:**

	*x*	*y*	*z*	*U*_iso_*/*U*_eq_	
C1	1.0983 (4)	0.3759 (3)	0.4418 (3)	0.0477 (8)	
C2	0.9888 (4)	0.2914 (3)	0.4161 (3)	0.0476 (8)	
H2	1.0080	0.2395	0.4604	0.057*	
C3	0.8478 (4)	0.2825 (3)	0.3235 (3)	0.0425 (7)	
C4	0.8150 (3)	0.3595 (2)	0.2533 (3)	0.0381 (7)	
C5	0.9338 (3)	0.4458 (3)	0.2854 (3)	0.0425 (7)	
C6	1.0703 (4)	0.4550 (3)	0.3766 (3)	0.0476 (8)	
H6	1.1439	0.5133	0.3952	0.057*	
C7	0.7338 (4)	0.1967 (3)	0.3078 (3)	0.0481 (8)	
H7	0.7579	0.1559	0.3637	0.058*	
C8	0.4970 (4)	0.0922 (3)	0.2368 (3)	0.0589 (10)	
H8A	0.5537	0.0514	0.2923	0.071*	
H8B	0.4444	0.0402	0.1617	0.071*	
C9	0.3798 (5)	0.1534 (4)	0.2797 (4)	0.0736 (12)	
H9A	0.3209	0.1008	0.2975	0.088*	
H9B	0.4353	0.2075	0.3526	0.088*	
C10	0.2674 (4)	0.2124 (3)	0.1965 (4)	0.0615 (10)	
H10A	0.2108	0.1588	0.1235	0.074*	
H10B	0.1945	0.2418	0.2310	0.074*	
C11	0.4185 (5)	0.3916 (3)	0.2747 (4)	0.0718 (12)	
H11A	0.4459	0.4545	0.2522	0.108*	
H11B	0.5092	0.3668	0.3234	0.108*	
H11C	0.3523	0.4114	0.3175	0.108*	
C12	0.2176 (4)	0.3474 (3)	0.0846 (4)	0.0714 (12)	
H12A	0.1471	0.3782	0.1197	0.107*	
H12B	0.1642	0.2892	0.0163	0.107*	
H12C	0.2623	0.4036	0.0624	0.107*	
C13	0.2718 (3)	0.0922 (2)	−0.0969 (3)	0.0392 (7)	
C14	0.1551 (4)	0.0039 (3)	−0.1298 (3)	0.0444 (8)	
C15	0.0298 (4)	−0.0161 (3)	−0.2317 (3)	0.0491 (8)	
H15	−0.0441	−0.0741	−0.2496	0.059*	
C16	0.0137 (4)	0.0506 (3)	−0.3085 (3)	0.0473 (8)	
C17	0.1211 (4)	0.1372 (3)	−0.2818 (3)	0.0468 (8)	
H17	0.1089	0.1814	−0.3338	0.056*	
C18	0.2487 (3)	0.1596 (3)	−0.1770 (3)	0.0402 (7)	
C19	0.3602 (4)	0.2499 (3)	−0.1558 (3)	0.0410 (7)	
H19	0.3446	0.2842	−0.2165	0.049*	
C20	0.5820 (4)	0.3734 (2)	−0.0702 (3)	0.0426 (8)	
H20A	0.6134	0.4327	0.0007	0.051*	
H20B	0.5278	0.4038	−0.1360	0.051*	
C21	0.7228 (4)	0.3244 (3)	−0.0862 (3)	0.0492 (8)	
H21A	0.7857	0.3812	−0.0948	0.059*	
H21B	0.7822	0.3021	−0.0158	0.059*	
C22	0.6870 (4)	0.2261 (3)	−0.1911 (3)	0.0518 (9)	
H22A	0.7819	0.2021	−0.1975	0.062*	
H22B	0.6342	0.1659	−0.1788	0.062*	
C23	0.5051 (5)	0.1534 (3)	−0.3889 (4)	0.0679 (11)	
H23A	0.4458	0.1732	−0.4598	0.102*	
H23B	0.4381	0.1203	−0.3605	0.102*	
H23C	0.5744	0.1020	−0.4044	0.102*	
C24	0.6821 (5)	0.3107 (3)	−0.3452 (4)	0.0685 (11)	
H24A	0.7578	0.2658	−0.3609	0.103*	
H24B	0.7318	0.3779	−0.2880	0.103*	
H24C	0.6164	0.3272	−0.4163	0.103*	
Co1	0.52925 (4)	0.23544 (3)	0.08521 (3)	0.03191 (14)	
N1	0.6028 (3)	0.1710 (2)	0.2245 (2)	0.0474 (7)	
N2	0.3391 (3)	0.3031 (2)	0.1696 (3)	0.0528 (7)	
N3	0.4787 (3)	0.2878 (2)	−0.0621 (2)	0.0394 (6)	
N4	0.5922 (3)	0.2520 (2)	−0.3004 (2)	0.0474 (7)	
O1	0.6877 (2)	0.35591 (18)	0.16610 (19)	0.0471 (5)	
O2	0.3892 (2)	0.10552 (17)	−0.00081 (19)	0.0468 (5)	
S1	0.90029 (11)	0.54412 (8)	0.20420 (9)	0.0586 (3)	
H1	0.8663	0.4994	0.1023	0.088*	
S2	1.27446 (11)	0.38591 (10)	0.55553 (9)	0.0675 (3)	
H2A	1.2950	0.2982	0.5781	0.101*	
S3	0.17582 (12)	−0.08143 (8)	−0.03595 (9)	0.0622 (3)	
H3	0.2279	−0.0276	0.0638	0.093*	
S4	−0.14439 (11)	0.02070 (9)	−0.43967 (9)	0.0659 (3)	
H4	−0.1689	0.1019	−0.4757	0.099*	

### Atomic displacement parameters (Å^2^)

**Table:**

	*U*^11^	*U*^22^	*U*^33^	*U*^12^	*U*^13^	*U*^23^
C1	0.0326 (17)	0.059 (2)	0.0439 (19)	0.0020 (16)	0.0116 (15)	0.0034 (16)
C2	0.0425 (19)	0.056 (2)	0.0388 (18)	0.0019 (16)	0.0090 (15)	0.0121 (16)
C3	0.0371 (17)	0.0453 (18)	0.0431 (18)	−0.0015 (14)	0.0141 (15)	0.0084 (15)
C4	0.0344 (17)	0.0406 (17)	0.0392 (17)	0.0012 (13)	0.0147 (14)	0.0079 (14)
C5	0.0349 (17)	0.0433 (18)	0.049 (2)	−0.0014 (14)	0.0176 (15)	0.0065 (15)
C6	0.0309 (17)	0.049 (2)	0.056 (2)	−0.0069 (15)	0.0172 (16)	−0.0008 (17)
C7	0.048 (2)	0.049 (2)	0.046 (2)	−0.0007 (16)	0.0118 (17)	0.0204 (16)
C8	0.057 (2)	0.057 (2)	0.060 (2)	−0.0175 (18)	0.0081 (19)	0.0317 (19)
C9	0.074 (3)	0.090 (3)	0.066 (3)	−0.021 (2)	0.032 (2)	0.030 (2)
C10	0.053 (2)	0.065 (2)	0.074 (3)	−0.0110 (19)	0.037 (2)	0.012 (2)
C11	0.077 (3)	0.064 (3)	0.075 (3)	−0.013 (2)	0.045 (2)	−0.007 (2)
C12	0.059 (3)	0.073 (3)	0.092 (3)	0.017 (2)	0.037 (2)	0.024 (3)
C13	0.0336 (17)	0.0399 (17)	0.0416 (18)	−0.0004 (13)	0.0131 (14)	0.0070 (14)
C14	0.0403 (18)	0.0389 (18)	0.052 (2)	−0.0026 (14)	0.0164 (16)	0.0085 (15)
C15	0.0365 (18)	0.047 (2)	0.059 (2)	−0.0058 (15)	0.0186 (16)	0.0028 (17)
C16	0.0332 (17)	0.056 (2)	0.047 (2)	0.0004 (15)	0.0132 (15)	0.0055 (16)
C17	0.0379 (18)	0.058 (2)	0.0435 (19)	0.0015 (16)	0.0138 (15)	0.0138 (16)
C18	0.0342 (17)	0.0443 (18)	0.0424 (18)	0.0008 (14)	0.0150 (14)	0.0104 (14)
C19	0.0378 (18)	0.0455 (18)	0.0426 (19)	0.0020 (14)	0.0159 (15)	0.0148 (15)
C20	0.0462 (19)	0.0381 (17)	0.0417 (18)	−0.0084 (14)	0.0138 (15)	0.0113 (14)
C21	0.0410 (19)	0.053 (2)	0.054 (2)	−0.0061 (16)	0.0169 (16)	0.0169 (17)
C22	0.048 (2)	0.054 (2)	0.060 (2)	0.0040 (17)	0.0252 (18)	0.0183 (18)
C23	0.074 (3)	0.063 (3)	0.063 (3)	−0.011 (2)	0.028 (2)	0.005 (2)
C24	0.079 (3)	0.066 (3)	0.071 (3)	−0.008 (2)	0.038 (2)	0.022 (2)
Co1	0.0278 (2)	0.0329 (2)	0.0329 (2)	−0.00619 (16)	0.00689 (17)	0.01176 (17)
N1	0.0420 (16)	0.0451 (16)	0.0528 (17)	−0.0079 (13)	0.0119 (14)	0.0179 (13)
N2	0.0532 (18)	0.0510 (17)	0.0589 (19)	−0.0032 (14)	0.0285 (15)	0.0107 (15)
N3	0.0354 (14)	0.0402 (15)	0.0440 (16)	−0.0024 (12)	0.0149 (13)	0.0128 (12)
N4	0.0480 (17)	0.0489 (17)	0.0499 (17)	−0.0031 (13)	0.0230 (14)	0.0134 (13)
O1	0.0384 (13)	0.0505 (13)	0.0474 (13)	−0.0090 (10)	0.0072 (10)	0.0174 (11)
O2	0.0438 (13)	0.0453 (13)	0.0446 (13)	−0.0062 (10)	0.0060 (10)	0.0152 (10)
S1	0.0502 (5)	0.0510 (5)	0.0719 (7)	−0.0105 (4)	0.0145 (5)	0.0242 (5)
S2	0.0368 (5)	0.0930 (8)	0.0551 (6)	−0.0024 (5)	0.0001 (4)	0.0128 (5)
S3	0.0628 (6)	0.0505 (6)	0.0667 (6)	−0.0164 (5)	0.0108 (5)	0.0243 (5)
S4	0.0386 (5)	0.0860 (7)	0.0545 (6)	−0.0090 (5)	0.0002 (4)	0.0098 (5)

### Geometric parameters (Å, °)

**Table:**

C1—C2	1.368 (4)	C14—S3	1.733 (3)
C1—C6	1.397 (5)	C15—C16	1.389 (5)
C1—S2	1.743 (3)	C15—H15	0.9300
C2—C3	1.402 (4)	C16—C17	1.371 (4)
C2—H2	0.9300	C16—S4	1.742 (3)
C3—C4	1.420 (4)	C17—C18	1.398 (4)
C3—C7	1.440 (4)	C17—H17	0.9300
C4—O1	1.297 (4)	C18—C19	1.447 (4)
C4—C5	1.426 (4)	C19—N3	1.287 (4)
C5—C6	1.367 (4)	C19—H19	0.9300
C5—S1	1.740 (3)	C20—N3	1.486 (3)
C6—H6	0.9300	C20—C21	1.525 (4)
C7—N1	1.280 (4)	C20—H20A	0.9700
C7—H7	0.9300	C20—H20B	0.9700
C8—N1	1.467 (4)	C21—C22	1.519 (5)
C8—C9	1.533 (6)	C21—H21A	0.9700
C8—H8A	0.9700	C21—H21B	0.9700
C8—H8B	0.9700	C22—N4	1.460 (4)
C9—C10	1.533 (5)	C22—H22A	0.9700
C9—H9A	0.9700	C22—H22B	0.9700
C9—H9B	0.9700	C23—N4	1.462 (4)
C10—N2	1.479 (4)	C23—H23A	0.9600
C10—H10A	0.9700	C23—H23B	0.9600
C10—H10B	0.9700	C23—H23C	0.9600
C11—N2	1.465 (5)	C24—N4	1.448 (4)
C11—H11A	0.9600	C24—H24A	0.9600
C11—H11B	0.9600	C24—H24B	0.9600
C11—H11C	0.9600	C24—H24C	0.9600
C12—N2	1.475 (5)	Co1—O2	1.922 (2)
C12—H12A	0.9600	Co1—O1	1.925 (2)
C12—H12B	0.9600	Co1—N1	2.003 (3)
C12—H12C	0.9600	Co1—N3	2.011 (2)
C13—O2	1.290 (4)	S1—H1	1.2000
C13—C18	1.422 (4)	S2—H2A	1.2000
C13—C14	1.429 (4)	S3—H3	1.2000
C14—C15	1.367 (5)	S4—H4	1.2000
			
C2—C1—C6	120.4 (3)	C16—C17—C18	120.6 (3)
C2—C1—S2	120.4 (3)	C16—C17—H17	119.7
C6—C1—S2	119.2 (3)	C18—C17—H17	119.7
C1—C2—C3	120.4 (3)	C17—C18—C13	121.0 (3)
C1—C2—H2	119.8	C17—C18—C19	117.8 (3)
C3—C2—H2	119.8	C13—C18—C19	121.2 (3)
C2—C3—C4	121.4 (3)	N3—C19—C18	127.0 (3)
C2—C3—C7	117.2 (3)	N3—C19—H19	116.5
C4—C3—C7	121.3 (3)	C18—C19—H19	116.5
O1—C4—C3	124.6 (3)	N3—C20—C21	110.5 (2)
O1—C4—C5	120.2 (3)	N3—C20—H20A	109.6
C3—C4—C5	115.2 (3)	C21—C20—H20A	109.6
C6—C5—C4	123.3 (3)	N3—C20—H20B	109.6
C6—C5—S1	119.1 (2)	C21—C20—H20B	109.6
C4—C5—S1	117.7 (2)	H20A—C20—H20B	108.1
C5—C6—C1	119.4 (3)	C22—C21—C20	114.2 (3)
C5—C6—H6	120.3	C22—C21—H21A	108.7
C1—C6—H6	120.3	C20—C21—H21A	108.7
N1—C7—C3	126.6 (3)	C22—C21—H21B	108.7
N1—C7—H7	116.7	C20—C21—H21B	108.7
C3—C7—H7	116.7	H21A—C21—H21B	107.6
N1—C8—C9	110.0 (3)	N4—C22—C21	112.0 (3)
N1—C8—H8A	109.7	N4—C22—H22A	109.2
C9—C8—H8A	109.7	C21—C22—H22A	109.2
N1—C8—H8B	109.7	N4—C22—H22B	109.2
C9—C8—H8B	109.7	C21—C22—H22B	109.2
H8A—C8—H8B	108.2	H22A—C22—H22B	107.9
C10—C9—C8	117.0 (3)	N4—C23—H23A	109.5
C10—C9—H9A	108.0	N4—C23—H23B	109.5
C8—C9—H9A	108.0	H23A—C23—H23B	109.5
C10—C9—H9B	108.0	N4—C23—H23C	109.5
C8—C9—H9B	108.0	H23A—C23—H23C	109.5
H9A—C9—H9B	107.3	H23B—C23—H23C	109.5
N2—C10—C9	114.7 (3)	N4—C24—H24A	109.5
N2—C10—H10A	108.6	N4—C24—H24B	109.5
C9—C10—H10A	108.6	H24A—C24—H24B	109.5
N2—C10—H10B	108.6	N4—C24—H24C	109.5
C9—C10—H10B	108.6	H24A—C24—H24C	109.5
H10A—C10—H10B	107.6	H24B—C24—H24C	109.5
N2—C11—H11A	109.5	O2—Co1—O1	173.14 (10)
N2—C11—H11B	109.5	O2—Co1—N1	89.21 (10)
H11A—C11—H11B	109.5	O1—Co1—N1	90.08 (10)
N2—C11—H11C	109.5	O2—Co1—N3	90.29 (9)
H11A—C11—H11C	109.5	O1—Co1—N3	89.40 (9)
H11B—C11—H11C	109.5	N1—Co1—N3	171.48 (11)
N2—C12—H12A	109.5	C7—N1—C8	116.5 (3)
N2—C12—H12B	109.5	C7—N1—Co1	125.1 (2)
H12A—C12—H12B	109.5	C8—N1—Co1	118.3 (2)
N2—C12—H12C	109.5	C11—N2—C12	109.4 (3)
H12A—C12—H12C	109.5	C11—N2—C10	112.0 (3)
H12B—C12—H12C	109.5	C12—N2—C10	108.0 (3)
O2—C13—C18	124.4 (3)	C19—N3—C20	115.0 (3)
O2—C13—C14	120.1 (3)	C19—N3—Co1	124.1 (2)
C18—C13—C14	115.6 (3)	C20—N3—Co1	120.83 (19)
C15—C14—C13	122.9 (3)	C24—N4—C22	111.6 (3)
C15—C14—S3	119.1 (2)	C24—N4—C23	110.9 (3)
C13—C14—S3	118.0 (2)	C22—N4—C23	111.7 (3)
C14—C15—C16	119.5 (3)	C4—O1—Co1	128.2 (2)
C14—C15—H15	120.2	C13—O2—Co1	128.09 (19)
C16—C15—H15	120.2	C5—S1—H1	109.5
C17—C16—C15	120.5 (3)	C1—S2—H2A	109.5
C17—C16—S4	121.0 (3)	C14—S3—H3	109.5
C15—C16—S4	118.5 (3)	C16—S4—H4	109.5

## References

[bb1] Allen, F. H., Kennard, O., Watson, D. G., Brammer, L., Orpen, A. G. & Taylor, R. (1987). *J. Chem. Soc. Perkin Trans. 2*, pp. S1–19.

[bb2] Enraf–Nonius (1989). *CAD-4 Software* Enraf–Nonius, Delft, The Netherlands.

[bb3] Harms, K. & Wocadlo, S. (1995). *XCAD4* University of Marburg, Germany.

[bb4] North, A. C. T., Phillips, D. C. & Mathews, F. S. (1968). *Acta Cryst.* A**24**, 351–359.

[bb5] Sheldrick, G. M. (2008). *Acta Cryst.* A**64**, 112–122. 10.1107/S0108767307043930 18156677

[bb6] Shi, L., Fang, R.-Q., Xue, J.-Y., Xiao, Z.-P., Tan, S.-H. & Zhu, H.-L. (2008). *Aust. J. Chem.* **61**, 288–296.

[bb7] Xu, S.-P., Shi, L., Lv, P.-C., Fang, R.-Q. & Zhu, H.-L. (2009). *J. Coord. Chem.* **62**, 2048–2057.

